# Diet quality and associated factors in Brazilian undergraduates during the COVID-19 pandemic

**DOI:** 10.3389/fnut.2023.1169147

**Published:** 2023-05-24

**Authors:** Liana L. P. Galvão, Thanise S. S. Santos, Betzabeth Slater, Clélia de O. Lyra, Priscilla M. Rolim, Alanderson A. Ramalho, Tatiane Dalamaria, Fernanda Andrade Martins, Doroteia A. Höfelmann, Sandra P. Crispim, Bartira M. Gorgulho, Paulo R. M. Rodrigues, Dirce M. Marchioni, Bruna L. L. Maciel

**Affiliations:** ^1^Health Sciences Postgraduate Program, Nutrition Department, Federal University of Rio Grande do Norte, Natal, RN, Brazil; ^2^Nutrition Interventions Research Group, School of Nursing, Federal University of Minas Gerais, Belo Horizonte, MG, Brazil; ^3^Faculty of Public Health, Center for Epidemiological Research in Nutrition and Health (NUPENS), University of São Paulo, São Paulo, SP, Brazil; ^4^Nutrition Department, School of Public Health, University of São Paulo, São Paulo, SP, Brazil; ^5^Graduate Program in Public Health, Federal University of Acre, Rio Branco, AC, Brazil; ^6^Health and Sports Science Center, Nutrition Course, Federal University of Acre, Rio Branco, AC, Brazil; ^7^Food and Nutrition Postgraduate Program, Nutrition Department, Federal University of Parana, Curitiba, PR, Brazil; ^8^Department of Food and Nutrition, Federal University of Mato Grosso, Nutrition Faculty, Cuiabá, MT, Brazil

**Keywords:** nutrition, perceived stress, food security, sleep assessment, SARS-CoV-19

## Abstract

**Background:**

Diet quality is associated with psychobiological, psychological, biological, and physiological factors of individuals, and in the context of prolonged stress, such as the COVID-19 pandemic, it can lead to a worsening of the quality of food for undergraduates. This study aimed to analyze diet quality and associated factors in Brazilian undergraduates.

**Methods:**

Data were collected from 4,799 undergraduate students from all Brazilian regions, from August 2020 to February 2021. The online questionnaire contained socioeconomic variables, the ESQUADA scale to assess diet quality, self-referred changes in weight, the Brazilian food insecurity scale (EBIA), sleep assessment and the perceived stress scale. Unconditional multiple logistic regression analysis was performed to study variables associated with poor and very poor diet quality.

**Results:**

Most of participants presented a good diet quality (51.7%), while 9.8% had a poor or very poor diet quality and only 1.1% had an excellent diet quality. 58.2% of undergraduates reported to have an increase in weight during the pandemic and 74.3% of the students presented elevated stress during the pandemic. Logistic regressions showed students who gained weight during the pandemic presented the highest AOR = 1.56 (95% CI = 1.12–2.20) for poor or very poor diet quality. The elevated perceived stress was also associated with a higher AOR = 2.85 (95% CI = 1.71–4.74) for poor or very poor diet quality.

**Conclusion:**

Most of the studied undergraduates presented a good diet quality. Nevertheless, poor/very poor diet quality was associated with higher perceived stress and weight gain. Our study indicated that policies should beaimed at the socioeconomically most vulnerable undergraduates, those in a situation of food and nutritional insecurity, high perceived stress, and who gained weight during the pandemic.

## 1. Introduction

Adequate nutrition is a fundamental aspect of promoting and protecting health, as it determines the health conditions of individuals, in addition to being consistently associated with chronic diseases that affect the world population (1). Also, diet quality is related to better health conditions, immunity, and coping with diseases (2–5).

Studies have shown that undergraduate students are more prone to nutritionally unbalanced diets because of the student routine, living alone, having few cooking skills, or food insecurity (6–8). Undergraduates tend to have food choices that are easier to acquire and/or prepare, which, on the other hand, are foods with a higher energy density (6, 9). Thus, evaluating diet quality is important in undergraduates. Also, many university students depend on meals offered by University Restaurants, which had their activities suspended during the COVID-19 lockdowns (10) The validated diet quality scale (ESQUADA) evaluates diet in a broader context, including the consumption of fresh, minimally processed, and ultra-processed foods and dietary practices (such as cooking, and substituting meals for snacks) (11). Thus, using this instrument could give important light on assessing diets and policy planning for undergraduates.

Eating habits and behaviors can be changed due to sociobiological, psychological, biological and physiological factors (12), and studies have shown that situations with a context of prolonged stress, such as the COVID-19 pandemic, impact all these factors (13, 14). In addition, social distancing caused changes in the behavior of individuals (15), and eating behavior was also affected by the economic and social crisis associated with the health emergency (16).

The restrictions caused by the COVID-19 pandemic may have committed to maintaining of a healthy and varied diet, reducing the consumption of fresh foods such as fruits, vegetables and fish, increasing the preference for ready-to-eat and ultra-processed foods rich in fats, sugars and salt (17). Furthermore, studies show that psychological and emotional responses to social distancing can increase the risk of developing dysfunctional eating behaviors; because people may be more likely to seek rewards and gratifications physiologically associated with food consumption to contrast and respond to the negative experience associated with increased stress (18, 19).

The literature is quite limited considering the assessment of undergraduates’ diet quality, especially during the COVID-19 pandemic. However, with the closing of university restaurants, the transition to remote classes, and social, economic, and mental changes, there is a need to understand undergraduate students’ diet quality and associated factors in the context of a health emergency. Therefore, this study aimed to assess the diet quality and associated factors in undergraduates from Brazilian universities. The hypothesis under study was that undergraduates’ poorer diet quality was associated with worse nutritional status, food insecurity, altered sleep duration, and higher perceived stress.

## 2. Methods

### 2.1. Ethics

Each center approved the study protocol from local institutional ethics review boards at: the Federal University of Acre (CAAE 36814320.9.0000.5010, #4.267.655), Federal University of Rio Grande do Norte (CAAE: 35918620.7.0000.5292, #4.391.606), Federal University of Mato Grosso (CAAE 36582820.0.0000.8124, #4.242.364), University of São Paulo (CAAE 36402820.9.0000.5421, #4.232.859), and the Federal University of Paraná (CAAE 36250320.2.0000.0102, #4.256.436). All participants recorded consent online to participate in the study.

### 2.2. Study design and participants

This is a cross-sectional study, with data collection from August 2020 to February 2021, corresponding to half of the first wave and the beginning of the second wave of COVID-19 in Brazil. This study is part of a multicenter project carried out at universities in five different states, covering the five regions of Brazil: Acre, Rio Grande do Norte, Mato Grosso, São Paulo and Paraná. The project is entitled “Food insecurity, nutritional status and lifestyle in the academic community during the COVID-19 pandemic – BRAZUCA COVID” and was carried out at the Federal University of Acre – UFAC, Federal University of Rio Grande do Norte – UFRN, Federal University of Mato Grosso – UFMT, University of São Paulo – USP, and the Federal University of Paraná – UFPR. Students regularly matriculated in undergraduate courses in public universities were eligible for the study. During the data collection period, the number of students enrolled in the studied universities were: 31,911 at UFAC, 38,478 at UFRN, 15,891 at UFMT, 59,779 at USP, and 29,406 at UFPR.

An online questionnaire was created on the Google Forms platform and sent to the students’ institutional emails. The questionnaire was a compilation of socioeconomic variables (including sex, race/color, self-referred changes in weight, family income, and income change during the pandemic) and the Diet Quality Scale (ESQUADA) scale to assess diet quality (11), the Brazilian food insecurity scale (EBIA) (20), sleep assessment (21) and the perceived stress scale (22), totaling 108 questions. The sample was of convenience with non-probabilistic sampling.

### 2.3. Diet quality assessment

The diet quality scale (ESQUADA) was applied to assess the students’ diet quality (11). The ESQUADA originally presents 25 items on dietary practices (such as replacing meals with snacks and the habit of cooking) and the consumption of fresh, minimally processed, and ultra-processed food. It evaluates diet quality, not measuring the nutritional value but other important aspects of food, bringing a more global view of diet quality that is not just nutritional. The scale items present alternative answers covering frequency, location, and food consumed. The items on the ESQUADA include polytomous and dichotomous responses, but all are ordinal; they have categories ordered according to alignment with the worst or best quality of the diet (11).

The ESQUADA presented better accuracy in the continuum of diet quality between scores −2 and + 2, confirmed goodness-of-fit (RMSEA = 0.01; SRMSR = 0.02; CIF = 0.99 and TLI = 0.99), and adequate empirical reliability (0.70). In addition, differential behavior was not identified for any item, considering age and sex (11). For validation of the ESQUADA scale, the item response theory (IRT) analyses was used, which allows the selection of items from the larger set of questions without jeopardizing the score estimate, according to the IRT’s principle of invariance (23). Thus, considering the use of the IRT to validate the ESQUADA, 15 items from the scale were selected for the present study. Items with more discrimination of diet quality, more participation in the description of ESQUADA levels, and no similarity with questions already included in the online questionnaire due to content or placement of the scale were selected.

The ESQUADA score scale was constructed by grouping into levels indicative of the cumulative trait of diet quality. The scores estimated on a scale with a mean equal to 0 and standard deviation equal to 1 were analyzed and categorized into five levels of diet quality: “very poor” (scores ≤ −2); “poor” (scores > −2 and ≤ −1), “good” (scores > −1 and ≤ 0,5); “very good” (scores >0,5 and ≤ 2,5); and “excellent” (scores >2,5) (11).

### 2.4. Self-referred changes in weight

Self-referred changes in weight during the pandemic were registered in the online form as a categorical variable (no; yes, for less; yes, for more; I do not wish to inform).

### 2.5. Food insecurity assessment

Food insecurity was assessed using the adapted and validated Brazilian food insecurity scale (EBIA) (Cronbach’s alpha = 0.91) (24, 25). This scale measures food insecurity by evaluating the influence of money scarcity on food availability and consumption of adults and children living in the house. The EBIA’s application and analysis have demonstrated that there are common aspects across different sociocultural contexts of food insecurity represented in the scale, including the (1) psychological component – anxiety or doubt about the future availability of food in the house to meet the needs of the locals; (2) food quality – impairment of socially established preferences about foods and its variety at home; (3) quantitative reduction of food among adults; (4) quantitative reduction in children’s diet; and (5) hunger – when someone does not eat all day due to lack of money to buy foods (26–28).

The scale is based on the sum of positive responses to 14 polytomous questions. Scores are organized within cutoff points equivalent to graded theoretical constructs on food security: food security (total score = 0), mild food insecurity (in households with people <18 years old, total score from 1 to 5; households without people <18 years old, total score 1 to 3), moderate food insecurity (in households with children under 18 years old, total score 6 to 9; households without people under the age of 18 years old, total score 4 to 5) and severe food insecurity (in households with people <18 years old, total score 10–14; households without people <18 years old, total score 6–8).

### 2.6. Sleep assessment

Sleep assessment was performed through the questions: “On a weekday, what time do you usually sleep?,” “On a weekday, what time do you usually wake up?,” “On weekends, what time do you usually wake up?,” “What time do you usually sleep?” and “On weekends, what time do you usually wake up?” before and during the pandemic.

Sleep duration was estimated by the weighted average of sleep time during the week (difference between bedtime and waking up during the week) and at the weekend (difference between bedtime and waking up on the weekend), using the equation: [(weekday sleep duration x 5) + (weekend sleep duration x 2)]/7 (21).

### 2.7. Perceived stress

Perceived stress was evaluated using the perceived stress scale – PSS (29), a version with 10 polytomous items, validated for the Brazilian population (Cronbach’s alpha = 0.86) (22) and categorized as mild (scores ≤13), moderate (scores between 14 and 19), and high (scores ≥20) (30). It is a self-reported measure to assess the degree to which situations in a person’s life are classified as stressful.

### 2.8. Statistical analysis

The statistical analysis was performed using the Statistical Package for the Social Sciences SPSS®, version 11.5 (SPSS Inc. Chicago, IL) and Graph Pad Prism version 3.0 (Graph Pad Software, San Diego, CA).

The normality of the quantitative variables was tested using the Kolmogorov–Smirnov test to present data as means (SD) or medians (Q1 – Q3). Categorical variables were presented through frequency distribution, and associations were evaluated using the Chi-square test. For quantitative data without normal distributions, the Kruskal-Wallis test and the Dunns *post hoc* test were used. Given the large size of the studied sample, the significance level was set at 1% to avoid type 1 errors.

Primarily, bivariate analysis explored the effect of a single variable on diet quality assessment with the unadjusted odds ratios (OR) and their respective 95% confidence intervals (95% CI). Then, logistic regression models were calculated, considering the dichotomized ESQUADA classification as a dependent variable (1 = very poor /poor diet quality; 0 = good / very good / excellent diet quality). The adjustment of the final model was guaranteed by observing the Omnibus test, with *p* values less than 0.05, and the Hosmer and Lemeshow test, considering p values greater than 0.05. Thus, as independent variables, the final model included self-reported changes in weight, food insecurity, sleep assessment during the weekend before the pandemic, and perceived stress. Sex, age and study site were used in the final model as adjustment variables. The adjusted odds ratios (AOR) and their respective 95% CI were presented, variables with Wald test’s *p* values <0.01 were those considered to significantly contribute in the model. The significance level was set at 1% to avoid type 1 errors, given the large sample size.

## 3. Results

**Figure 1:**
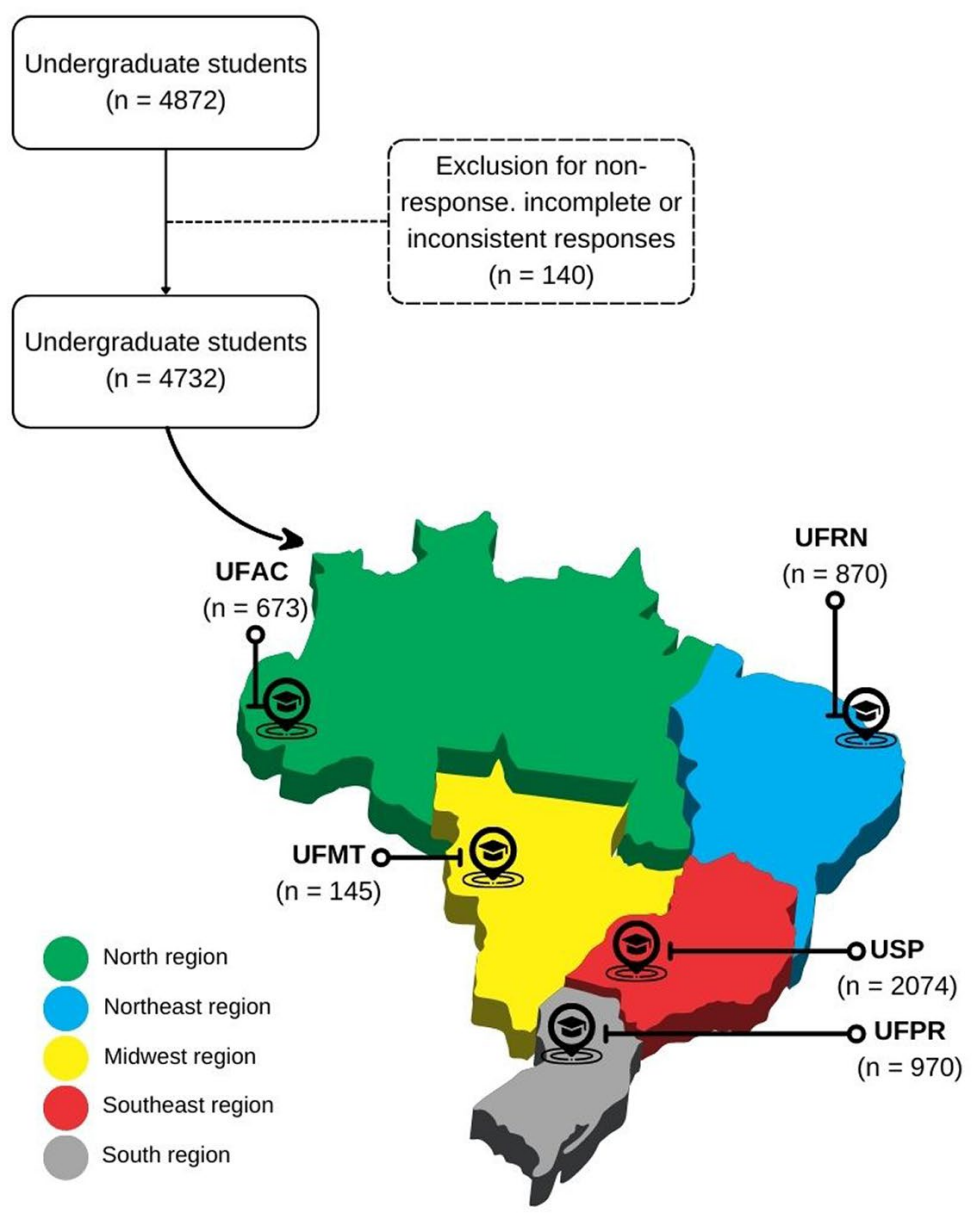
Flowchart of studied undergraduates (*n* = 4,732), considering the study sites. UFAC – Federal University of Acre, UFRN – Federal University of Rio Grande do Norte, UFMT – Federal University of Mato Grosso, USP – University of São Paulo, UFPR – Federal University of Paraná.

A total of 4,872 undergraduates of the studied public universities responded to the questionnaire; 140 responses were excluded from this study due to non-response, incomplete or inconsistent responses, totaling 4,732 participants. Of these, 673 were from UFAC, 870 from UFRN, 145 from UFMT, 2074 from USP, and 970 from UFPR (Figure 1).

The sociodemographic and eating characteristics of the undergraduates is shown in Table 1. The median age was 22.0 (20.0–26.0) years, and the questionnaire was mostly answered by women (66.1%). Family income was mainly around 1–6 minimum wages, and 47.9% of undergraduates reported a reduction in family income during the pandemic. Considering weight change, 47.9% of undergraduates had an increase in weight during the pandemic. Most undergraduates rated their health status before the pandemic as good or regular (74.9%), and only 4.7% of participants rated it as poor or very poor. On the other hand, during the pandemic, 24.4% of the students reported poor or very poor health conditions. The Brazilian food insecurity scale showed that 37.8% of students presented food insecurity, with 4.4% of students classified as severe. In addition, the pandemic’s sleeping time increased during the week and decreased on the weekend. The perceived stress showed that 74.3% of the students presented elevated stress during the pandemic (Table 1).

**Table 1:** Sociodemographic, eating characteristics and associated factors of the studied undergraduates (*n* = 4,799).

Characteristics	Total *n* (%) or median (Q1 – Q3)
	Median (Q1 – Q3)
Age	22.0 (20.0–26.0)
Sex	*n* (%)
Male	1,628 (33.9)
Female	3,171 (66.1)
Family income in minimum wages^a^	*n* (%)
None	132 (2.7)
0–1	708 (14.8)
1–3	1,473 (30.7)
3–6	1,040 (21.7)
6–9	536 (11.2)
9–12	332 (6.9)
12–15	218 (4.5)
>15	322 (6.7)
NI/NWI	38 (0.8)
Family income change during the pandemic	*n* (%)
No	1985 (41.4)
Yes, for less	2,302 (47.9)
Yes, for more	466 (9.7)
NI/NWI	46 (1.0)
Weight change during the pandemic	*n* (%)
No	596 (12.4)
Yes, for less	1,322 (27.6)
Yes, for more	2,670 (55.6)
NI/NWI	211 (4.4)
Health status before the pandemic	*n* (%)
Very good	980 (20.4)
Good	2,343 (48.9)
Regular	1,244 (26.0)
Poor	189 (3.9)
Very poor	37 (0.8)
Health status during the pandemic	*n* (%)
Very good	476 (10.0)
Good	1,407 (29.4)
Regular	1730 (36.2)
Poor	895 (18.7)
Very poor	270 (5.7)
Food security classification	*n* (%)
Food security	2,893 (60.3)
Mild food insecurity	1,242 (25.9)
Moderate food insecurity	360 (7.5)
Severe food insecurity	209 (4.4)
NI/NWI	95 (2.0)
Sleep assessment in minutes	Median (Q1 – Q3)
Weed during pandemic	480 (420–600)
Weekend during pandemic	510 (450–570)
Week before the pandemic	450 (390–500)
Weekend before the pandemic	540 (480–600)
Perceived stress	*n* (%)
Low	436 (9.1)
Moderate	797 (16.6)
Elevated	3,566 (74.3)

a The minimum wage in Brazil is R$ 1,100, around $ 212. NI/NWI: Not informed/did not wish to inform.

**Figure 2:**
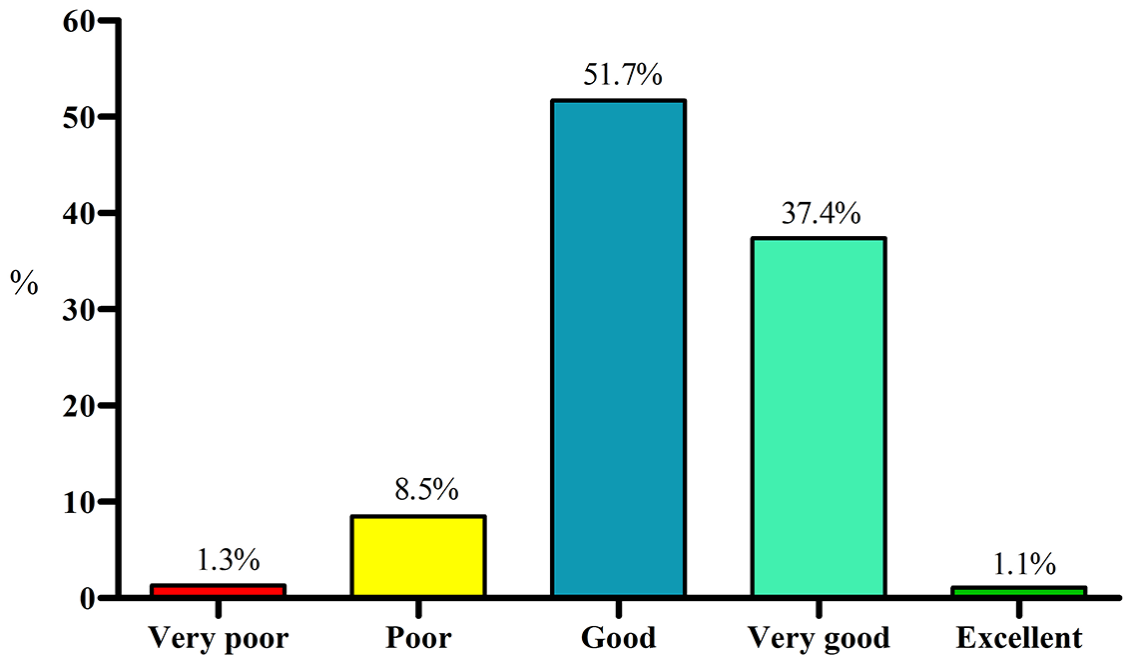
Diet quality assessment in undergraduates (*n* = 4,799), assessed using the diet quality scale (ESQUADA). Chi-square test, *p* < 0.001.

Considering diet quality, according to the answers provided by the undergraduates, 9.8% of the students were classified as having a poor or very poor diet quality, while 51.7% had a good diet quality and only 1.1% had an excellent diet (Figure 2). The bivariate association analysis (Table 2) showed that most undergraduates with very poor or poor diet quality increased weight during the pandemic (Chi-square test, *p* < 0.001). None of the undergraduates with severe food insecurity in their households presented excellent diet quality (Chi-square test, p < 0.001). In addition, undergraduates with poor or very poor diet quality had an elevated perceived stress, 82.8 and 84.8%, respectively (Chi-square test, *p* < 0.001; Table 2).

**Table 2:** Diet quality of undergraduates (*n* = 4,799) according to anthropometric, food variables, sleep and stress during the pandemic.

Variable	Total *N* (%)	Very poor *N* (%)	Poor *N* (%)	Good *N* (%)	Very good *N* (%)	Excellent *N* (%)	Chi-square test *p* value
Weight change during the pandemic							
No	596 (13.0)	4 (6.6)	38 (9.5)	284 (11.9)	262 (15.5)	8 (15.7)	<0.001
Yes, for less	1,322 (28.8)	14 (23.0)	77 (19.3)	618 (25.9)	587 (34.7)	26 (51.0)	
Yes, for more	2,670 (58.2)	43 (70.5)	284 (71.2)	1,483 (62.2)	843 (49.8)	17 (33.3)	
Total	4,588 (100.0)	61 (100.0)	399 (100.0)	2,385 (100.0)	1,692 (100.0)	51 (100.0)	
Variable	Total *N* (%)	Very poor *N* (%)	Poor *N* (%)	Good *N* (%)	Very good *N* (%)	Excellent *N* (%)	Chi-square test *p* value
Food security classification							
Food security	2,893 (61.5)	30 (48.4)	233 (57.7)	1,401 (57.4)	1,189 (68.1)	40 (81.6)	<0.001
Mild food insecurity	1,242 (26.4)	22 (35.5)	113 (28.0)	712 (29.2)	388 (22.2)	7 (14.3)	
Moderate food insecurity	360 (7.7)	5 (8.1)	32 (7.9)	224 (9.2)	97 (5.6)	2 (4.1)	
Severe food insecurity	209 (4.4)	5 (8.1)	26 (6.4)	105 (4.3)	73 (4.2)	0 (0.0)	
Total	4,704 (100.0)	62 (100.0)	404 (100.0)	2,442 (100.0)	1747 (100.0)	49 (100.0)	
Variable	Total Median (Q1 – Q3)	Very poor Median (Q1 – Q3)	Poor Median (Q1 – Q3)	Good Median (Q1 – Q3)	Very good Median (Q1 – Q3)	Excellent Median (Q1 – Q3)	Kruskal-Wallis test *p* value
Sleep assessment in minutes							
Week during pandemic	480 (420–540)	510 (420–540)	510 (420–540)	510(420–540)	510(420–540)	510(420–540)	0.504
Weekend during pandemic	510 (450–570)	540 (480–600)	525 (457–570)	510 (450–570)	510(450–550)	480(472–540)	0.036
Week before the pandemic	450 (390–500)	450 (420–532)	450 (400–510)	450 (390–510)	450 (390–480)	445(412–480)	0.345
Weekend before the pandemic	540 (480–600)	540 (480–600)	540 (480–600)	540 (480–600)	540 (480–600)	495^a^ (450–540)	<0.001
Variable	Total *N* (%)	Very poor *N* (%)	Poor *N* (%)	Good *N* (%)	Very good *N* (%)	Excellent N (%)	Chi-square test *p* value
Perceived stress							
Low	436 (9.1)	1 (1.6)	17 (4.1)	170 (6.8)	235 (13.2)	13 (25.0)	<0.001
Moderate	797 (16.6)	10 (15.6)	47 (11.4)	391 (15.7)	336 (18.9)	13 (25.0)	
Elevated	3,566 (74.3)	53 (82.8)	350 (84.5)	1929 (77.5)	1,208 (67.9)	26 (50.0)	
Total	4,799 (100.0)	64 (100.0)	414 (100.0)	2,490 (100.0)0	1779 (100.0)	52 (100.0)	

a Dunns post hoc test indicated *p* < 0.001 when compared to the other groups.

Logistic regressions (Table 3) further explored the observed associations, where students who gained weight during the pandemic had an AOR = 1.59 (95% CI = 1.13–2.24) for poor or very poor diet quality. In addition, elevated perceived stress was also associated with diet quality with an AOR = 2.89 (95% CI = 1.74–4.82) for poor or very poor diet quality.

**Table 3:** Logistic regressions for variables associated with poor or very poor diet quality in Brazilian undergraduates during the COVID-19 pandemic.

Independent variables	ESQUADA classification			
	OR (95% CI)	*p* value	AOR (95% CI)	*p* value
Weight change during the pandemic				
No	−		−	
Yes, for less	0.98 (0.67–1.43)	0.896	0.86 (0.58–1.26)	0.855
Yes, for more	1.84 (1.32–2.57)	<0.001	1.59 (1.13–2.24)	0.008
Food security classification				
Food security	−		−	
Mild food insecurity	1.22 (0.98–1.52)	0.076	1.11 (0.88–1.39)	0.376
Moderate food insecurity	1.15 (0.79–1.65)	0.463	0.98 (0.67–1.44)	0.930
Severe food insecurity	1.74 (1.16–2.60)	0.007	1.45 (0.94–2.23)	0.088
Sleep assessment in minutes				
Weekend before the pandemic	1.00 (0.99–1.01)	0,887	1.00 (0.99–1.01)	0.609
Perceived stress				
Low	−		−	
Moderate	1.78 (1.04–3.08)	0.036	1.81 (1.03–3.18)	0.038
Elevated	2.96 (1.82–4.79)	<0.001	2.89 (1.74–4.82)	<0.001

*ESQUADA: Diet quality scale. The ESQUADA classification was dichotomized to become a dependent variable (1 = very poor/poor diet quality; 0 = good/very good/excellent diet quality). OR: crude odds ratio, from bivariate analysis; AOR: adjusted odds ratio, considering all variables in the model. Sex, age and study site were included as adjustment variables.

## 4. Discussion

This study aimed to assess the diet quality and associated factors in undergraduates from Brazilian universities. Most of the studied undergraduates presented good diet quality. This result might be explained by the fact that undergraduates might have given more attention to food preparation during lockdowns and that some went back to their parents’ homes, away from the university campus, to reduce expenses with housing and food (31, 32), thus having more assistance to prepare/consume a diet with good quality. Nevertheless, a considerable number of undergraduates had poor or very poor diet quality, which was associated with weight gain during the pandemic and perceived stress. Regarding the latter, it was notable that most undergraduates rated their perceived stress as very high during the pandemic.

Undergraduates who gained weight also demonstrated poor or very poor diet quality. In the ESQUADA scale, poor or very poor diet quality are the levels with more frequent consumption of ultra-processed foods (11, 33). Studies have shown that consuming foods with high energy density, such as ultra-processed and fast foods, is associated with weight change in undergraduates (34, 35). In addition, increased consumption of ultra-processed foods and weight gain can increase the incidence of chronic noncommunicable diseases (36). Furthermore, there was an increase in food prices in the Brazilian market, mainly for staple foods such as rice, beans, vegetables, fruits, and meats, which already preceded the pandemic and intensified even more (37). In March 2021, the value of the basic food basket in Brazil cost around R$ 626 (around $ 121), showing an increase of 20.7% compared to March 2020. Therefore, with the pandemic’s social and economic impacts and the increase in staple foods price, purchasing power worsened, especially for the most vulnerable families, leading to an increase in the consumption of processed and ultra-processed foods, which with the increase in the price of staple foods, are becoming increasingly cheaper (38), further affecting the diet quality of the undergraduates.

In Brazil, since 2012, with the enactment of the Quota Law (39), which reserves vacancies for undergraduate courses for students in public schools or who have a monthly income *per capita* ≤1.5 minimum wages (the minimum wage in Brazil is R$ 1,100, around $ 212) or who declare themselves black, brown, or indigenous, there was an increase in the inclusion of socio-demographically vulnerable students in university (40), which may corroborate for a lower purchasing power of Brazilian undergraduates.

Our research has shown that undergraduates with high perceived stress are 2.9 times more likely to have poor or very poor diet quality. Stress is a condition that threatens a person’s physical and emotional well-being and prevents progress, so that the person to react physiologically, behaviorally, cognitively, and emotionally to that state of stress (41).

Thus, searching for energy and sugar rich diets that activate the pleasure zones is related to a stress-coping strategy (42). A study in the United Kingdom applied a food frequency questionnaire and the perceived stress scale in 3,706 undergraduates. In the study, the greater consumption of sweets, snacks, and fast foods was significantly associated with a greater perception of stress in female undergraduates, and a reduction in the consumption of fruits, salads, and cooked vegetables was observed in referred stressful situations in both female and male (43).

Some studies have shown that with the social distancing measures during the pandemic, there was a reduction in the consumption of healthier foods and an increase in higher energy foods in undergraduates around the world, even causing binge eating associated with high-stress situations and anxiety (14, 44–46). In Brazil, it was no different, in adults aged between 18 and 29 there was a proportional increase in the consumption of unhealthy foods during the lockdown (47). With social distancing, Brazilian universities experienced the transition from face-to-face to remote learning, where students saw the need to study inmates in their homes. As a result, undergraduates mentioned that the restrictions of the pandemic caused an increase in stress and anxiety (48).

The shift to remote learning impacted undergraduates. Dash et al. (49) observed high psychological distress in tertiary students during the pandemic. In the study, better diet quality was also significantly correlated with fewer symptoms of psychological distress. The increased perceived stress in our study might be explained by reduced social interactions and access to student residency services, increasing academic and financial pressures, and altering the lifestyles of these undergraduates (49, 50). Increased stress has been associated with increased intake of foods with high energy density, rich in sugars and saturated fats, and reduced vegetable intake (44–46), which indicates poorer diet quality. This fact might explain that, in our study, most undergraduates with very poor or poor diet quality increased weight during the pandemic.

Although the logistic regression models did not demonstrate an association of poorer diet quality with food insecurity in our study, it is not possible to completely refute the initial hypothesis (that undergraduates’ poorer diet quality was associated with food insecurity), considering that the bivariate analysis showed an association of students with the poorest diet quality presenting food insecurity in their households during the pandemic. Also, in a study carried out at the University of Michigan (51) students who were in a situation of food insecurity presented a higher consumption of added total sugars, sugary drinks and a lower intake of fruits, vegetables, and fiber, when compared to undergraduates with food security. Other previous studies conducted in the United States of America have also demonstrated an association between poor or very poor diet quality and food insecurity status in undergraduates (52, 53). Furthermore, in previous analyses, considering the same population (8), undergraduates with a better diet quality were less likely to be inserted into a context of food and nutritional insecurity. In the present study, none of the undergraduates with severe food insecurity in the household presented an excellent diet quality.

Protecting the poorest groups from poor diet quality involves keeping food prices stable so they can access more nutritious food regularly. However, with the economic and political crisis that worsened during the pandemic, such action has become increasingly tricky (37), leading to global increased food insecurity. In this sense, Brazil is back on the hunger map. Within 2 years of the pandemic, the number of Brazilians who have nothing to eat has almost doubled, totaling 33.1 million (54), 14 million more Brazilians compared to 2020 (55). Our data showed that 47.9% of undergraduates had a reduction in family income during the pandemic, which may corroborate the restriction in food purchasing power in undergraduates’ households. Furthermore, greater food insecurity can act as a multiplier of the pandemic burden due to its negative consequences on health (56). Therefore, it is necessary to implement strategies aimed at guaranteeing access to food among the most vulnerable populations so that the impacts of the pandemic on health and nutrition can be mitigated (57). As an example of strategies that can be adopted, there are public policies aimed at ultra-processed foods, which involve regulation of the biochemical and nutritional composition of ultra-processed foods, products, and the drivers of food standards (58). Thus, the aim is to reduce consumption and the proportion of ultra-processed foods in countries with high consumption.

The factors associated with poor diet quality must be explored and understood because those most vulnerable undergraduates, even when eating at university restaurants, might have access to university cafeterias, where, in Brazil, purchasing healthy meals is more expensive, compromising diet quality (51). Moreover, this scenario may have been even more affected by the closing of university restaurants during the pandemic, where even if there were university scholarships for food aid that remained in the pandemic context, access to healthy foods rich in vitamins, fibers, and minerals was still more limited to students who lived in some degree of food insecurity.

Some limitations of the present study should be mentioned. First, the online data collection might have restricted the participation of those who did not have good access to the internet. However, contact with undergraduates took place through institutional email correspondence. In addition, in February 2021, when data collection ended, public universities already had support measures aimed at students, such as grants for acquiring internet and/or electronic equipment such as a notebook to ensure the monitoring of online classes for the most vulnerable. Furthermore, non-probabilistic sampling may have caused a selection bias because the motivation to participate in the research may have been greater in those undergraduates most affected by the pandemic. On the other hand, identifying and understanding these individuals were part of the research. Still, we were restricted to the students’ self-reported data. Therefore, there may be an underestimation or overestimation of the results.

The study’s strengths are the measurement of diet quality, food insecurity, and perceived stress concomitantly, allowing the observation of the demonstrated associations. In addition, data collection was conducted in universities in all Brazilian regions.We observed the diet quality of undergraduates in the face of a health emergency. Thus, it was possible to see which factors can associate with diet quality during the scenario of a pandemic. The results can contribute to the planning of public policies for this group of individuals, who may be experiencing social, economic, mental, and health difficulties due to the pandemic, which can directly and negatively impact education, in addition to preparing the public policies to face possible future scenarios of health emergencies such as a pandemic.

## 5. Conclusion

Our data showed that most of the studied undergraduates presented a good diet quality. Nevertheless, there was a considerable proportion of undergraduates with poor or very poor diet quality; and poor/very poor diet quality was associated with higher perceived stress and weight gain.

It is necessary to improve diet quality, especially for younger generations like most undergraduates. In Brazilian university students, our study indicated that policies should be directed to the most vulnerable, those in a situation of food and nutritional insecurity, high perceived stress, and who gained weight during the pandemic. Further studies should include food and nutrition education programs, access to healthy food in the studied population, and which factors lead to food choices in situations of increased stress and anxiety.

## Data availability statement

The raw data supporting the conclusions of this article will be made available by the authors upon request to the corresponding author.

## Ethics statement

The studies involving human participants were reviewed and approved by ethical review boards at: the Federal University of Acre (CAAE 36814320.9.0000.5010, #4.267.655), Federal University of Rio Grande do Norte (CAAE: 35918620.7.0000.5292, #4.391.606), Federal University of Mato Grosso (CAAE 36582820.0.0000.8124, #4.242.364), University of São Paulo (CAAE 36402820.9.0000.5421, #4.232.859), and the Federal University of Paraná (CAAE 36250320.2.0000.0102, #4.256.436). The patients/participants provided their written informed consent to participate in this study.

## Author contributions

DM, BM, TS, CL, PMR, BG, PRR, DH, SC, BS, and AR: designed research. LG and BM: analyzed data. LG, BM, CL, PMR, BG, PRR, TS, DH, SC, BS, TD, FAM, and AR: conducted research. DM: resources. LG and BM: wrote paper. CL, PMR, BG, PRR, TS, DH, SC, BS, AR, and DM: review and edited. DM: project administration and funding acquisition. All authors contributed to the article and approved the submitted version.

## Funding

This research was partially funded with resources from the Universal MCTI/CNPq No. 01/2016 grant (process No. 405837/2016–0), and was supported by the National Council for the Federal Agency for Support and Evaluation of Graduate Education in Brazil, the Coordination for the Improvement of Higher Education Personnel – CAPES (Coordenação de Aperfeiçoamento de Pessoal de Nível Superior), Ministry of Education of Brazil, Brasilia, DF, Brazil, for master’s scholarships.

## Conflict of interest

The authors declare that the research was conducted in the absence of any commercial or financial relationships that could be construed as a potential conflict of interest.

## Publisher’s note

All claims expressed in this article are solely those of the authors and do not necessarily represent those of their affiliated organizations, or those of the publisher, the editors and the reviewers. Any product that may be evaluated in this article, or claim that may be made by its manufacturer, is not guaranteed or endorsed by the publisher.

## Abbreviations

UFAC: Federal University of Acre
UFRN: Federal University of Rio Grande do Norte
UFMT: Federal University of Mato Grosso
USP: University of São Paulo
UFPR: Federal University of Paraná
ESQUADA: Diet Quality Scale
EBIA: Brazilian Food Insecurity Scale
PSS: Perceived Stress Scale.
